# Fatty Acids in the Eggs of Red King Crabs from the Barents Sea

**DOI:** 10.3390/ani14020348

**Published:** 2024-01-22

**Authors:** Alexander G. Dvoretsky, Fatima A. Bichkaeva, Nina F. Baranova, Vladimir G. Dvoretsky

**Affiliations:** 1 Murmansk Marine Biological Institute of the Russian Academy of Sciences (MMBI RAS), 183038 Murmansk, Russia; 2 N. Laverov Federal Center for Integrated Arctic Research of the Ural Branch of the Russian Academy of Sciences (FECIAR UrB RAS), 163000 Arkhangelsk, Russia.

**Keywords:** red king crab, *Paralithodes camtschaticus*, fatty acids, eggs, nauplius, metanauplius, Barents Sea

## Abstract

**Simple Summary:**

Seafood by-products contain a variety of valuable components, including bioactive peptides and essential fatty acids. The red king crab is a large, commercially important crustacean supporting profitable fisheries in the Barents Sea. In Norway, female red king crabs are included in the fishery. Large adult females carry egg masses, but red king crab eggs have not yet been studied for fatty acid content. In this paper, we provide information regarding the fatty acid profiles of red king crab eggs. We found a higher proportion of polyunsaturated fatty acids in comparison to saturated and monounsaturated fatty acids. Total pools of fatty acid did not differ significantly in terms of the stage of embryo development, female size, limb injury status, and habitat conditions. Individual comparisons, however, indicated significant differences for some fatty acids, providing evidence that they may play a role in physiological processes. Red king crab eggs may be considered a product with high nutritional value and are recommended for wide use in the food, pharmaceutical, and biomedical industries.

**Abstract:**

The red king crab, *Paralithodes camtschaticus*, was introduced into the Barents Sea where, after a period of 30 years of adaptation, it has established a new population. This population has been commercially exploited over the past two decades, supporting profitable fisheries in both Russia and Norway. Biochemical studies aimed at assessing fatty acid profiles have been conducted, focusing primarily on the edible parts of red king crabs. Only recently have by-products been included in this research. Capture of female red king crabs is prohibited in Russia but is allowed in Norway. The fatty acids of the egg masses carried by these females have not yet been studied. To fill this knowledge gap, we assayed the fatty acid composition of eggs using gas–liquid chromatography. Our results showed a predominance of polyunsaturated fatty acids, while the concentrations of saturated and monounsaturated fatty acids were similar. Multivariate comparisons showed no significant differences in fatty acid profiles in terms of egg developmental stage (nauplius vs. metanauplius), habitat conditions (soft vs. hard bottoms), female size class, or number of autotomized limbs. However, individual comparisons showed some differences in fatty acids, the most important being the lower content of docosahexaenoic acid in eggs at the metanauplius stage compared to eggs at the nauplius stage, which is likely due to its essential role in the development of red king crab embryos. The total fatty acid content (53.94 mg g^−1^) was 2–87 times higher in eggs than in other red king crab tissues, confirming the critical role that fatty acids play in maintaining physiological processes during vitellogenesis. The high content of essential fatty acids and an optimal omega-3-to-omega-6 ratio (4.9) suggest that red king crab eggs are a good product for a healthy diet and a valuable source for extracting essential fatty acids.

## 1. Introduction

The red king crab, *Paralithodes camtschaticus* (Tilesius, 1815), is a large crustacean species with a wide distribution range from British Columbia (Canada) to the North Pacific, southwest to Korea at 140° E, and north at approximately 170° E through the Bering Sea [1]. There are three distinct populations of this species occupying seabed locations from the intertidal region to the continental slope around the North Pacific [1]. Besides these native populations, a non-native population was intentionally introduced into the Barents Sea in the 1960s by Russian scientists to establish a new profitable resource for the local fisheries in that region where no commercial crab stocks existed at that time [2]. After a 30-year period of adaptation and range expansion, this species had successfully formed a new self-sustaining population in the Barents Sea [2]. Further population growth and range extension both eastward and westward along the coastline of the Barents Sea supported a significant increase in the abundance and biomass of this species. By the late 2000s, the abundance had become sufficient to develop a local king crab industry, leading to the initiation of commercial fishing for red king crab in 2002 in Norway and 2004 in Russia [3]. In Russian waters, the population has demonstrated significant biomass fluctuations, mainly due to inadequate fishing pressure over the first decade and changes in the climate regime over the second decade of exploration [3]. However, the annual catch rates of red king crabs have increased from 10,820 t in 2020 to 11,629 t in 2021 and to 12,529 t in 2022 [3].

Red king crabs have been extensively researched, focusing on their distribution patterns, population dynamics [4], growth and reproduction [2,3], behavior and migrations [5], feeding and competition with native fauna [6,7,8], and symbiotic relationships [9,10], as well as fishery, management, and conservation aspects [3,4,11]. Biochemical assays have been conducted to describe their hormonal profiles in relation to life-history traits [12,13] and the nutritional quality and chemical composition of their edible parts [14]. Important by-products, such as the hepatopancreas [15] and shell [16], are used for the production of chitin, chitosan, α-glucosidase inhibitors, and the anticancer agent prodigiosin, as well as proteolytic enzymes and non-protein components such as essential fatty acids [17]. Recently, the fatty acid composition of other by-products, such as hemolymph and heart muscles, which are currently discarded at sea after standard onboard processing, has also been investigated [18,19].

Eggs are another red king crab product that is not typically processed, largely due to a lack of information on their biochemical composition and poor adaptation of laboratory protocols to assess conditions at capture [17]. In Russia, the fishing of female red king crabs is prohibited, which includes the harvesting of their eggs [3]. However, Norwegian authorities have established a regime that involves the harvesting of female red king crabs, with a quota of 100–120 t per year in a quota-regulated area. Furthermore, for maintaining a sustainable red king crab population, the harvest of female red king crabs is unrestricted within an open-access fishing area established in Norwegian waters west of 26° E, to prevent further westward and southward expansion of the red king crab population [20]. In Russia, red king crab roe is also consumed by tourists and local fishermen in Russia as a delicacy and exotic product. Female red king crabs are considered a potential for aquaculture, for conservation and scientific purposes, and, to some degree, for the food industry.

Lipids and fatty acids are important components in the development of crustaceans and are considered essential to their growth and success in molting [21]. Previous studies have shown that fatty acids in crustacean eggs can provide crucial energy stores for later developmental stages, and can provide valuable information on environmental factors affecting natural populations [22,23]. Furthermore, these findings can assist in the development of reliable nutritional protocols for cultivated species in hatchery settings [24,25].

This study aims to explore the fatty acid profiles of red king crab eggs in the Barents Sea region, with the objective of improving our understanding of the nutritional quality of red king crab by-products. The hypothesis is that the fatty acid profiles of the eggs will differ in relation to developmental stage, female size, number of injured limbs, and benthic habitats.

## 2. Materials and Methods

Our study was undertaken in Dalnezelenetskaya Bay, a small gulf with a total area of 2.23 km^2^ located on the Eastern Murman coast of the Kola Peninsula [26]. A more detailed description of our study site is available in our previous publications [27,28]. In July 2016, a total of 37 egg-bearing female red king crabs were collected at depths ranging from 8 to 32 m by scientific SCUBA divers familiar with the nearshore habitats of the coastal Barents Sea. Previous investigations have established that sample sizes of 3 to 15 crustaceans are adequate to characterize patterns in fatty acid compositions across different developmental stages of eggs [23,25,29,30].

When capturing the crabs, divers registered temperature, depth, and the type of benthic habitat where each female was collected. In accordance with information obtained during diving surveys, the seabed locations in the study area were divided into two categories: hard bottoms—habitats composed mainly of rock, boulders, outcroppings, and algal kelps—and soft bottoms—habitats where sand alone or in different combinations with pebbles and shells were present. Female red king crabs were delivered to the coastal laboratory for biological analysis [31], which included visual inspection for the egg category, shell condition, and presence/absence of injured legs. Each female was measured for carapace length (CL, the greatest straight-line distance from the posterior margin of the right eye orbit to the medial-posterior margin of the carapace) using a vernier caliper to the nearest 0.1 mm, and weighed on an electronic balance to the nearest 1 g. All crabs collected had light coxa, spines, dactyls, and ventral surface of exoskeleton, and their legs were full of muscle tissue, i.e., characteristics indicative of new shells (2–12 months post ecdysis) [31].

After biological analysis, two portions of eggs were removed from each egg clutch and analyzed. One subsample (10–15 g) was frozen and used for fatty acid analyses, while the other (1–2 g) was fixed in Bouin solution and examined under a stereo-microscope to determine the stage of embryo development. Eggs were separated with diluted bleach, placed between 2 transparent sheets and photographed under a digital camera. Egg diameter was measured using ImageView 4.11 software [32], after adjustments and calibrations, with 15 eggs randomly selected from each subsample for photographic documentation. The development of red king crab embryos includes five stages: cleavage (I), gastrula (II), nauplius (III), metanauplius (IV), and late-stage zoeal egg (V) [33].

In the laboratory of the Federal Center for Integrated Arctic Research (Arkhangelsk), fatty acids were extracted, and fatty acid methyl esters were prepared according to the method of Folch et al. [34] with modifications [14]. The homogenized sample was dissolved into 10 mL of an extracting chloroform–methanol mixture and a solution of nonadecanoic acid in chloroform. The resulting solution was mixed for 30 min and then held in a thermostat for 10–12 h at 25 °C for lipid extraction. The solution was then filtered and mixed with a chloroform–methanol (2:1) solution to achieve the final sample volume of 15 mL. A 0.74% water solution of CaCl_2_ (3 mL) was added to the sample and then stored in a refrigerator for 12 h. After stratification, the top layer containing water-soluble impurities was removed, while the lower layer was mixed with methanol and then evaporated to dryness using a vacuum evaporator Multivapor P12 (pressure 318 mbar, temperature 50 °C). The evaporated extract was added to 0.2 mL of the chloroform–methanol mixture, mixed for 5 min, and dissolved in a 1.5% solution of H_2_SO_4_ in methanol (2 mL). The sample was incubated in a water bath for 30 min at 90 °C. The sample was then placed in 0.8 mL of distilled water and incubated at ambient temperature for 2–4 h. The top fraction was pipetted into a 2 mL vial and evaporated again. The solution of fatty acid methyl esters (200 μL) was injected into an Agilent 7890A gas chromatograph (Agilent Technologies Inc., Wilmington, DE, USA) equipped with a flame ionization detector. The gas–liquid chromatography conditions were as follows: capillary column: Agilent DB-23 (60 m × 0.25 mm × 0.15 μm); carrier gas: nitrogen (injection volume 1 mm^3^) with a 1 mL min^−1^ flow rate; injection port temperature: 270 °C; flame ionization detector temperature: 280 °C; the column was programmed from an initial temperature of 130 °C (0.5 min hold), rising to 170 °C at 8.5 °C min^−1^, 206 °C at 2 °C min^−1^, 220 °C at 0.7 °C min^−1^, and 230 °C at 6 °C min^−1^. Fatty acid methyl esters were identified by comparing their retention times with those of Nu Chek Prep Inc. (Elysian, MN, USA) 569 standards in the Agilent Chem Station B.04.03 software.

Gas–liquid chromatography is an established technique utilized for analyzing the types and quantities of lipids in foods. This method is regarded as highly sensitive, generating results with high accuracy and reproducibility. Although gas–liquid chromatography is time-consuming, labor-intensive, and necessitates experienced and adequately trained personnel to perform, it remains extensively employed to identify and quantify fatty acids in crustacean eggs [23,25,29,30,35,36,37].

A principal component analysis (PCA) was used to identify underlying patterns in egg fatty acid composition and to simplify data presentation and interpretation. The Bray–Curtis dissimilarity index was used to perform PERMANOVA with 9999 permutations on the raw data to investigate differences in fatty acid composition between two egg stages (violet and brown), three female size classes (121–133 mm, 134–146 mm, and >146 mm CL), three groups of females with different numbers of injured limbs (0, 1, and >1), and two different types of benthic communities (soft and hard). Differences in individual fatty acid concentrations were assessed using one-way analysis of variance (ANOVA) followed by Tukey’s multiple comparison tests for normal data and the Kruskal–Wallis test (KWT) for non-normal data followed by Bonferroni multiple comparison tests. The Shapiro–Wilk test and modified Levene’s test were applied to examine normal distribution and homoscedasticity of data. When necessary, the data were transformed to satisfy normality and homogeneity assumptions. ANOVA was used to evaluate differences in egg diameter between the two developmental stages, and a chi-squared test was used to compare the percentage occurrence of females with violet and brown eggs. Pearson correlation coefficients were calculated for log-transformed data to identify potential associations between fatty acid content in the eggs and ovaries of red king crabs. Ovary fatty acid content data for the same females used in the egg fatty acid analysis were extracted from our previous publication [19]. Statistical analyses were carried out using NCSS PASS 2004 and PAST 3.26.

## 3. Results

The female red king crabs used for biochemical assays ranged from 121.5 to 162.4 mm in CL and from 934 to 2548 g in body weight (Table 1).

**Figure 1:**
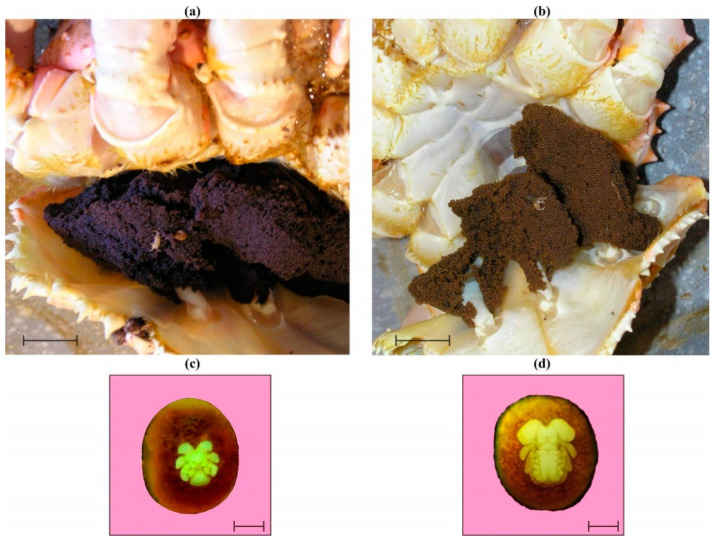
Eggs and developmental stages of red king crabs. (**a**)—violet eggs, scale bar 20 mm; (**b**)—brown eggs, scale bar 20 mm; (**c**)—nauplius stage, scale bar 300 µm; (**d**)—metanauplius stage, scale bar 300 µm.

Visual observations indicated that 33 females had uneyed violet eggs (Figure 1a), while the remaining 4 individuals had uneyed brown eggs (Figure 1b). 

However, stereo-microscope observations revealed that 28 of the 33 violet-egg clutches were at the nauplius stage (Figure 1c). The eggs from the remaining 5 violet-egg clutches and from all brown-egg clutches were at the metanauplius stage (Figure 1d) as defined and described by Nakanishi [33], Stevens [38], and Matyushkin [39]. Thus, the proportions of females with eggs at the nauplius and metanauplius stages were 75.7% and 24.3%, respectively. These percentages were significantly different from each other (χ^2^ = 8.53, *p* = 0.004). Additionally, egg diameter of nauplius-staged eggs (range, 850–1012 µm; mean ± SD, 939 ± 42 µm) was significantly lower than that of metanauplius-staged eggs (947–1049 µm; mean ± SD, 998 ± 26 µm) (ANOVA, F = 237.52, *p* < 0.001). Females with different-staged eggs had similar CL (ANOVA, F = 1.06, *p* = 0.310) and mean body weight (ANOVA, F = 1.12, *p* = 0.298).

Biochemical analysis detected 43 fatty acids in the eggs of red king crabs (Table 2).

Saturated fatty acids (SFAs) were mainly composed of palmitic (C16:0) and stearic (C18:0) acids. The mean levels of C16:0 were 8119 μg g^−1^ (15.2%) in nauplius-staged eggs and 7571 μg mL^−1^ (15.2%) in metanauplius-staged eggs, while for C18:0, these values accounted for 2505 μg g^−1^ (4.7%) and 2488 μg g^−1^ (5.0%), respectively. Oleic acid (C18:1n9C) was the predominant monounsaturated fatty acid (MUFA) with mean levels of 6987 μg g^−1^ (13.0%) in nauplius-staged eggs and 6685 μg g^−1^ (13.3%) in metanauplius-staged eggs, followed by palmitoleic acid (C16:1C) with mean levels of 3723 μg g^−1^ (6.9%) in nauplius-staged eggs and 3618 μg g^−1^ (7.2%) in metanauplius-staged eggs. Polyunsaturated fatty acids (PUFAs) were the dominant fatty acid types in the egg profiles of red king crabs. Arachidonic acid (C20:4n6) was the major n-6 PUFA, accounting for 2717 μg g^−1^ (5.0%) in nauplius-staged eggs and 2513 μg g^−1^ (5.0%) in metanauplius-staged eggs. 

The most prevalent n-3 polyunsaturated fatty acids (PUFAs) were eicosapentaenoic acid (EPA, C20:5n3) and docosahexaenoic acid (DHA, C22:6n3). EPA constituted 13,568 μg g^−1^ (25.1%) and 12,954 μg g^−1^ (25.8%) in nauplius-staged and metanauplius-staged eggs, respectively. The mean levels of DHA were 8537 μg g^−1^ (13.8%) in nauplius-staged eggs and 7007 μg g^−1^ (14.0%) in metanauplius-staged eggs. The percentage of PUFA content was significantly higher than that of saturated fatty acids (SFAs) and monounsaturated fatty acids (MUFAs) in both nauplius- (ANOVA, F = 4309.06, *p* < 0.001) and metanauplius-staged eggs (KWT, H = 20.22, *p* < 0.001). In contrast, pairwise comparisons showed insignificant differences between the proportions of SFAs and MUFAs (*p* > 0.05 in both cases).

**Figure 2:**
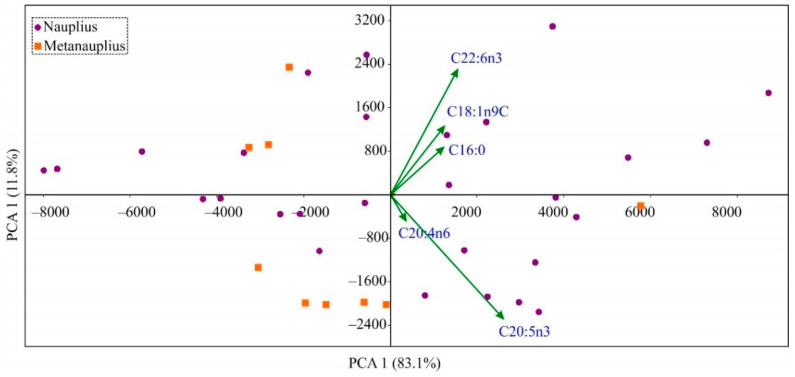
Principal component analysis (PCA) of the fatty acid composition of red king crab eggs in Dalnezelenetskaya Bay, July 2016.

A PCA biplot of the fatty acid data for red king crab eggs showed that principal components 1 and 2 (PC1 and PC2) cumulatively explained a majority of the variance in the data (94.9%) (Figure 2). 

The first axis separated females with eggs containing higher (positive scores) and lower (negative scores) concentrations of DHA and EPA. Almost all females with metanauplius-staged eggs were positioned on the left side of the biplot. However, many points representing females with nauplius-staged eggs also had negative PC1 scores, resulting in overlap between the two groups and insignificant differences in the fatty acid profiles between the different-staged eggs (PERMANOVA, F = 0.53, *p* = 0.533). Comparisons conducted for individual fatty acids confirmed the results of the PCA; a significantly lower value of DHA was found in metanauplius-staged eggs compared to nauplius-staged eggs (ANOVA, F = 4.66, *p* = 0.039). Additionally, significant differences were found for C9:0 and C:15 (ANOVA, *p* < 0.05), but these fatty acids had very low contributions to the total content.

A multivariate comparison revealed that the fatty acid profiles of females of different sizes were similar (PERMANOVA, F = 0.72, *p* = 0.517), except for three cases (C18:2n6t, C22:2, and C22:4n6), where significant differences were recorded (ANOVA or KWT, *p* < 0.05) with lower values found in the intermediate size class (134–146 mm CL) (Figure S1). The number of autotomized limbs did not affect the fatty acid profiles in the eggs (Table S1, PERMANOVA, F = 0.72, *p* = 0.517), but some less important fatty acids demonstrated significant variations. For C8:0, intact females had a higher concentration than females with injured legs (ANOVA, F = 4.98, *p* = 0.013), whereas the highest concentrations of C9:0, C13:0, C15:0, C17:0, and C22:5n6 were found in the eggs of females with one autotomized leg (ANOVA, *p* < 0.05) (Figure S2). Fatty acid profiles in the eggs of females captured on soft and hard bottoms did not differ significantly (PERMANOVA, F = 0.38, *p* = 0.642), and individual comparisons also showed insignificant results (Table S2, ANOVA or KWT, *p* < 0.05). Correlation coefficients indicated no significant difference between the concentration of SFAs, MUFAs, PUFAs, and total fatty acids in ovaries [19] and corresponding levels in the eggs (k = –0.014 … –0.059, *p* = 0.728–0.935).

## 4. Discussion

Fatty acids are crucial components of lipid-containing molecules such as triacylglycerols, fats, and waxes, and due to their high caloric content they serve as important chemical feedstocks in basic metabolic processes [40]. In crustacean invertebrates, the importance of fatty acids has been shown to increase significantly in response to high energy requirements during key physiological processes such as molting, limb regeneration, and especially mating and spawning [21,41,42].

Red king crab embryos develop inside eggs and have no external food sources. Therefore, the total fatty acid concentration in eggs (combined data for violet and brown eggs, 53,940 μg g^−1^) is higher than in hemolymph (617 μg g^−1^), leg muscles (2930 μg g^−1^), cardiac muscles (5240 μg g^−1^), ovaries (21,990 μg g^−1^), and even hepatopancreases of females (25,590 μg g^−1^) [14,15,18,19]. It is known that in decapods, dietary lipids accumulate in the hepatopancreas and are transferred to the ovary, and then to hatching eggs during the annual reproductive cycle [43,44]. Embryo development in red king crabs lasts 11–11.5 months [3]. Thus, these high fatty acid concentrations in the eggs are necessary to ensure the developmental processes during this period. Similar variations in the fatty acid content in different tissues with higher concentrations in eggs were reported for the blue swimmer crab *Portunus pelagicus* [23,45].

The fatty acid profiles in the red king crab eggs were dominated by PUFAs, while the SFA and MUFA contents were similar. A predominance of PUFAs has also been reported for the eggs of the crabs *Maja brachydactyla* [46], *Chionoecetes opilio* [29], *Cancer setosus* [47], and *Charybdis japonica* [37]. Similar proportions of PUFAs and MUFAs have been found in embryos of the lobster *Nephrops norvegicus* [30]. In contrast, MUFAs were relatively more prevalent than PUFAs in the eggs of the crab species *Uca rapax* [48], *Armases cinereum* [49], *Eriocheir sinensis* [25], and *Shinkaia crosnieri* [22], and the shrimp species *Chorismus antarcticus*, *Notocrangon antarcticus*, and *Nematocarcinus lanceopes* [50]. SFA-dominated profiles have been observed for the eggs of the crabs *Uca annulipes* [51], *Uca inversa*, *Uca urvillei*, *Uca chlorophthalmus*, *Uca vocans* [35], and *Portunus pelagicus* [52,53]. A balanced SFA/MUFA/PUFA ratio has been observed in the blue crab *Callinectes sapidus* [36].

According to Donaldson and Byesrdorfes [31], stages I, II, and III are considered “uneyed violet eggs”, stage IV represents “uneyed brown eggs”, and stage V is “eyed brown” or “orange eggs”. However, results from previous studies [39] and our data indicate that this classification is not accurate. In the Barents Sea in July, the proportions of females with eggs at stages III and IV were reported to be 63% and 37%, respectively [39]. Our ratio of 76%:24% is slightly different from these values, likely due to differences in environmental conditions in our sampling location. It is known that after fertilization and deposition of eggs on the female’s abdomen, the development of the eggs to hatching depends on water temperature and requires a specific number of heat units (degree-days) [38,54]. The study by Matyushkin [39] was conducted in Ura Bay, where the water temperature is higher than in Dalnezelenetskaya Bay [27,55]. Red king females in that location may rear eggs under more suitable conditions, yielding a faster egg maturation rate. Mating of red king crabs in the Barents Sea can occur any time from late January to early June, with a peak during March–April [56,57]. Therefore, we can assume that females with eggs at stage III spawned 1–2 months earlier than females with eggs at stage II.

Despite the developmental events that occur in red king crab embryos from the nauplius stage to the metanauplius stage, which are accompanied by a significant increase in egg diameter, the fatty acid content in the eggs remained consistent across different stages (Figure 2). This pattern may be explained by the low contribution of fatty acids to bioenergetic investments during these stages of development. During the nauplius stage, a white blastodisc is formed, and the optic lobes and rudiments of the mandibles, antennules, antennae, labrum, and pleopods appear [38]. During the metanauplius stage, there is further development of all the cephalic appendages mentioned above, plus two pairs of maxillipeds and three pairs of maxillae, and protuberances from the thorax begin to appear [38]. These processes may have specific requirements, which could explain the significantly lower content of two minor and one major fatty acid (DHA) in the eggs. Further development of red king crab embryos leads to significant transformations, including elongation of the unsegmented telson, its stretching over the head, and segmentation. The embryo gradually outgrows the yolk, and by the eighth month, all major body parts have appeared [38]. The embryo then increases in size until hatching [33] when the egg lipid content is completely consumed [58]. 

Similar patterns of fatty acid concentrations by egg maturation have been reported for many crustacean species. In general, the fatty acid content in early-staged eggs (stages I and II) or intermediate-staged eggs (stages II and III) did not differ significantly [36,37,46,48,49,59]. However, as vitellogenesis progressed, the amounts of almost all important fatty acids decreased considerably [23,24,35,37,51,53]. In other species, there was a continuous decrease in the fatty acid contents throughout embryo development [29,47,60]. It should be noted that the egg-staging systems used for these species do not directly correspond to the classification proposed for red king crabs, but they do reflect significant changes in the embryo’s morphology and/or physiology.

We observed that the total pool of fatty acids in eggs was not significantly affected by female size or limb injury status. Therefore, the significant variations found for certain fatty acids may be related to their role in the autotomy process, which can significantly affect the physiology of red king crabs [41]. This phenomenon has been shown for the Chinese mitten crab, *Eriocheir sinensis*, for which changes in the ratio of specific fatty acids were observed in relation to autotomy [61]. Alternatively, the observed variations may also be attributed to changes in the physiological status of different-sized red king crab specimens, which could be associated with their different migration activity [5], or to differences in food habits among female red king crabs [58]. In our previous study, we found that the fatty acid profiles in hemolymph differed significantly between red king crabs captured on soft and hard sediments, indicating the role of feeding in determining the total pool of fatty acids [18]. Similar effects have been observed in other crab species under both natural [62] and laboratory conditions [63,64]. Our study also revealed insignificant dissimilarity between the fatty acid signatures of red king crab females from different habitats. This result is not surprising, as the females had already extruded their clutches of eggs well before being collected and were likely outside the study area at the time. Similarly, we found no significant correlations between the fatty acid contents in the ovaries and eggs. This can be explained by the fact that the current ovaries had no direct links to the previously extruded eggs.

In the Barents Sea, the nauplius stage of red king crabs occurs from May to September, peaking in July at 43%. The metanauplius stage occurs in almost equal proportions (37–40%) from July through September [39]. Given the tendency of fatty acid content to decrease during embryo development, as observed in other crab species, we can conclude that the harvest of red king crab females for high-quality eggs with high concentrations of essential fatty acids should be completed before October, when eggs of most females are at the late zoea stage. In Norway, the annual female quota for the 2023 fishery season is 120 t. Based on the mean weight of a female of 1550 g, the mean egg weight of 0.61 mg, and the mean weight of an egg clutch of 131.8 g, or 8.5% of the total female weight [57,65], we can estimate that Norwegian fishermen can receive 10.2 t of raw red king crab eggs.

It is well established that EPA and DHA are highly valuable compounds that can be obtained through consumption or extraction from seafood. These fatty acids have gained significant market value due to their associated beneficial health effects [66]. The consumption of seafood products and by-products containing long-chain n-3 polyunsaturated fatty acids (PUFAs) has been shown to have positive effects on human health, with demonstrated anticancer, antidepressant, anti-diabetic, antihyperglycemic, antihypertensive, anti-inflammatory, antimicrobial, antioxidant, anti-rheumatic, and immunomodulatory properties [67,68,69,70,71].

In accordance with the Joint Food and Agriculture Organization of the United Nations/World Health Organization Expert Consultation on Fats and Fatty Acids in Human Nutrition recommendations, the total daily intake of n-3 fatty acids can range between 0.5% and 2% of energy intake, with a minimum dietary requirement of alpha-linolenic acid (ALA, C18:3n3) of more than 0.5% of energy for adults to prevent deficiency-related symptoms. The higher value of 2% of energy intake for ALA in combination with EPA and DHA, i.e., 0.25–2.0 g per day, can be considered indicative of a healthy diet. For adult males and non-pregnant/non-lactating adult females, the recommended daily intake of EPA + DHA is 0.25 g. However, for adult pregnant and lactating females, the minimum daily intake for optimal adult health and fetal and infant development is 0.3 g EPA + DHA, of which at least 0.2 g should be DHA [72].

Due to their higher concentrations of fatty acids, particularly n-3 polyunsaturated fatty acids, and a well-balanced n-3/n-6 ratio in comparison to other tissues such as muscles, red king crab eggs can be recommended for direct human consumption.

## 5. Conclusions

Our study provides novel insights into the fatty acid composition of different-staged eggs of the commercially important red king crab (*Paralithodes camtschaticus*) inhabiting the Barents Sea. We detected high concentrations of fatty acids reaching 50–53 mg g^−1^. These results indicate that lipids play a critical role in sustaining embryonic development in the red king crab. We observed that the fatty acid profiles of eggs at different developmental stages were largely similar, with the exception of docosahexaenoic acid (DHA), whose levels were significantly reduced in eggs at the metanauplius stage. This suggests DHA has an important nutritional function for embryos undergoing the transition from the nauplius to metanauplius phase. Notably, the fatty acid profiles of red king crabs of varying sizes, with different numbers of autotomized appendages, and from either hard or soft benthic habitats, were mostly analogous, apart from minor variations in select fatty acids. Considering the demand for alternative sources of essential omega-3 fatty acids, our results provide valuable information demonstrating that red king crab eggs are a nutrient-dense source of essential fatty acids and have an optimal ratio of omega-3 to omega-6 PUFAs, rendering them an exceptional food product for human consumption and use in pharmaceutical and food industries.

## Figures and Tables

**Table 1:** Carapace length and weight of female red king crabs collected for egg masses in Dalnezelenetskaya Bay, July 2016.

Egg Stage	N	X	SD	Min	Max
	Carapace length, mm				
Nauplius	28	140.2	9.4	121.5	162.4
Metanauplius	9	147.0	6.5	137.0	157.0
Combined	37	139.4	8.8	121.5	162.4
	Weight, g				
Nauplius	28	1913	329	1317	2548
Metanauplius	9	1773	401	934	2098
Combined	37	1879	347	934	2548

Note. N—sample size, X—mean, SD—standard deviation, Min—minimum, Max—maximum.

**Table 2:** Fatty acid composition of different stages of red king crab eggs from Dalnezelenetskaya Bay, July 2016.

Fatty Acid	Level, μg g^−1^				Proportion, %			
	Nauplius		Metanauplius		Nauplius		Metanauplius	
	X ± SE	Min–Max	X ± SE	Min–Max	X ± SE	Min–Max	X ± SE	Min–Max
C6:0	1.8 ± 0.1	1.3–3.3	2.4 ± 0.4	1.1–4.2	0.002 ± 0	0–0.005	0.005 ± 0.001	0.002–0.008
C8:0	3.7 ± 0.2	2.1–5.6	3.4 ± 0.4	1.9–5.1	0.007 ± 0	0.004–0.011	0.007 ± 0.001	0.003–0.011
C9:0	3.3 ± 0.2	1.7–6.7	4.2 ± 0.4	1.8–5.8	0.006 ± 0.001	0.003–0.012	0.009 ± 0.001	0.003–0.013
C10:0	7.2 ± 0.4	4.5–11	7.6 ± 0.4	6.1–9.7	0.013 ± 0.001	0.009–0.022	0.015 ± 0.001	0.013–0.02
C11:0	3.7 ± 0.3	1.5–9.1	4.8 ± 0.6	2.7–8	0.007 ± 0.001	0.003–0.017	0.01 ± 0.001	0.005–0.016
C12:0	146 ± 6	84–207	147 ± 12	116–234	0.27 ± 0.01	0.19–0.36	0.29 ± 0.01	0.26–0.35
C13:0	21.2 ± 0.9	9.3–31.3	19.7 ± 1.4	13.2–25.8	0.039 ± 0.001	0.029–0.055	0.039 ± 0.002	0.029–0.049
C14:0	925 ± 37	480–1335	854 ± 60	534–1025	1.72 ± 0.04	1.45–2.31	1.71 ± 0.1	1.11–1.98
C15:0	491 ± 17	278–687	426 ± 13	364–499	0.92 ± 0.02	0.74–1.12	0.86 ± 0.02	0.74–0.94
C16:0	8119 ± 289	5453–12,450	7571 ± 338	6447–9969	15.2 ± 0.2	13.4–17.3	15.2 ± 0.4	14.1–17.1
C17:0	367 ± 12	242–478	329 ± 10	266–380	0.69 ± 0.01	0.55–0.84	0.66 ± 0.02	0.56–0.75
C18:0	2505 ± 92	1572–3468	2488 ± 91	2215–3081	4.7 ± 0.1	3.8–5.1	5 ± 0.1	4.5–5.3
C20:0	131 ± 7	51–199	141 ± 14	87–187	0.24 ± 0.01	0.16–0.35	0.28 ± 0.02	0.19–0.34
C21:0	36.8 ± 4.6	7.1–74.8	26.4 ± 7.1	10.7–73	0.065 ± 0.007	0.02–0.138	0.051 ± 0.012	0.024–0.112
C22:0	26.6 ± 2.5	5.8–59.5	33.6 ± 3.8	18.7–44.5	0.049 ± 0.004	0.012–0.104	0.067 ± 0.007	0.039–0.091
C23:0	102 ± 8	43–252	153 ± 41	53–448	0.19 ± 0.01	0.09–0.47	0.29 ± 0.06	0.11–0.66
C24:0	306 ± 31	91–697	185 ± 18	116–250	0.55 ± 0.05	0.23–1.03	0.37 ± 0.04	0.26–0.57
C14:1t	15.2 ± 0	15.2–15.2	4.1 ± 0	4.1–4.1	0.001 ± 0.001	0–0.033	0.001 ± 0.001	0–0.008
C14:1C	11.5 ± 0.9	3.2–28	11.1 ± 1.2	6–15.1	0.021 ± 0.002	0.006–0.06	0.022 ± 0.002	0.013–0.031
C15:1	5.2 ± 1.2	0.8–13.5	3.4 ± 0.1	3.1–3.6	0.004 ± 0.001	0–0.024	0.002 ± 0.001	0–0.007
C16:1t	95 ± 5	22–139	102 ± 6	82–133	0.17 ± 0.01	0–0.28	0.21 ± 0.01	0.17–0.28
C16:1C	3723 ± 155	2113–5142	3618 ± 237	2973–5350	6.9 ± 0.1	6.1–7.8	7.2 ± 0.1	6.6–7.9
C17:1	6.2 ± 0.7	2.9–14.6	7.9 ± 1.1	4.7–12.3	0.009 ± 0.001	0–0.021	0.014 ± 0.003	0–0.025
C18:1n9t	288 ± 11	191–413	264 ± 18	138–344	0.541 ± 0.016	0.383–0.709	0.529 ± 0.033	0.286–0.615
C18:1n9C	6987 ± 303	4266–11,360	6685 ± 385	5726–9485	13 ± 0.2	11.2–15.2	13.3 ± 0.3	12.5–15.5
C20:1	1114 ± 57	562–1897	1107 ± 82	784–1508	2.1 ± 0.1	1.4–3.4	2.2 ± 0.1	1.8–2.9
C22:1	105 ± 6	59–200	131 ± 15	83–205	0.2 ± 0.01	0.12–0.32	0.26 ± 0.03	0.18–0.42
C24:1	49.4 ± 2.8	24.9–83.2	49 ± 6	25.8–87	0.092 ± 0.005	0.049–0.152	0.096 ± 0.008	0.053–0.128
C18:2n6t	153 ± 25	13–424	106 ± 49	10–366	0.27 ± 0.04	0–0.87	0.23 ± 0.11	0.02–0.8
C18:2n6C	664 ± 29	336–995	574 ± 33	467–778	1.2 ± 0	1–1.5	1.1 ± 0	1–1.3
C18:3n3	344 ± 22	152–587	325 ± 39	215–605	0.63 ± 0.03	0.41–0.96	0.63 ± 0.04	0.48–0.89
C18:3n6	203 ± 14	86–341	172 ± 13	101–228	0.38 ± 0.02	0.18–0.62	0.35 ± 0.03	0.15–0.44
C20:2n6	620 ± 26	364–913	608 ± 37	472–747	1.2 ± 0	0.9–1.4	1.2 ± 0.1	1–1.5
C20:3n6	102 ± 6	56–216	108 ± 9	83–160	0.19 ± 0.01	0.13–0.34	0.22 ± 0.01	0.17–0.27
C20:4n6	2717 ± 129	1183–4251	2513 ± 210	1672–3349	5.1 ± 0.2	3.6–6.7	5 ± 0.4	3.7–6.8
C22:2n6	7 ± 0.9	0.9–22.6	5.9 ± 1.4	1.6–14	0.014 ± 0.002	0.002–0.042	0.012 ± 0.003	0.003–0.029
C20:5n3	13,568 ± 592	7530–18,394	12,954 ± 790	10,126–17,596	25.1 ± 0.4	21.2–28.7	25.8 ± 0.8	21–28
C22:6n3	8537 ± 372	5007–12,065	7007 ± 456	5715–9962	15.8 ± 0.3	13.1–19.1	14 ± 0.6	11.9–17
C20:3n3	178 ± 13	60–404	182 ± 22	98–270	0.33 ± 0.02	0.19–0.64	0.36 ± 0.04	0.22–0.49
C22:4n6	208 ± 11	136–415	177 ± 9	136–221	0.4 ± 0.02	0.24–0.86	0.36 ± 0.02	0.31–0.44
C22:3n3	6.3 ± 1	0.9–15.7	3.4 ± 1.2	1–11.1	0.011 ± 0.002	0–0.029	0.007 ± 0.003	0.002–0.025
C22:5n6	146 ± 6	83–215	122 ± 6	93–157	0.27 ± 0.01	0.2–0.4	0.24 ± 0.01	0.21–0.27
C22:5n3	849 ± 33	568–1237	831 ± 69	566–1162	1.6 ± 0.1	0.9–2.6	1.7 ± 0.2	1.3–2.5
∑SFA	13,194 ± 456	8369–18,916	12,730 ± 538	10,919–16,217	24.6 ± 0.2	23.1–26.6	25.5 ± 0.7	23.8–30.1
∑MUFA	12,377 ± 496	7304–18,425	11,976 ± 688	10,296–17,069	23 ± 0.3	20.5–25.7	23.9 ± 0.4	23–26.1
∑PUFA	28,296 ± 1092	15,831–37,468	25,684 ± 1252	22,454–34,507	52.4 ± 0.3	49.4–55.4	51.3 ± 0.5	48.2–52.7
Total	53,867 ± 2006	31,504–74,809	50,060 ± 2392	43,831–67,793	100 ± 0	100–100	100 ± 0	100–100
∑n-3	23,482 ± 936	13,534–32,105	21,301 ± 1096	18,908–29,395	–	–	–	–
∑n-6	4814 ± 198	2297–7057	4386 ± 245	3546–5316	–	–	–	–
∑n-9	8544 ± 350	5117–13,143	8236 ± 464	6998–11,567	–	–	–	–
∑n-7	3819 ± 157	2182–5268	3727 ± 241	3074–5487	–	–	–	–
n-3/n-6	4.9 ± 0.1	3.6–6.3	4.9 ± 0.2	3.9–5.8	–	–	–	–

Note. X—mean, SE—standard error, SFA—saturated fatty acids, MUFA—monounsaturated fatty acids, PUFA—polyunsaturated fatty acids.

## Data Availability

Data are contained within the article and Supplementary Materials.

## Supplementary Materials

The following supporting information can be downloaded at: https://www.mdpi.com/article/10.3390/ani14020348/s1, Figure S1. Significant variation in the content of fatty acids in the eggs of female red king crabs of different sizes from the Dalnezelenetskaya Bay, July 2016. Vertical bars show standard errors. Different letters show significant differences between groups; Figure S2. Significant variations in fatty acid content in eggs of female red king crabs with different numbers of injured legs in Dalnezelenetskaya Bay, July 2016. Vertical bars show standard errors. Different letters show significant differences between groups; Table S1. Fatty acid composition (μg g^−1^) of red king crab eggs in crabs with different numbers of injured legs (0, 1, and >1) in Dalnezelenetskaya Bay, July 2016. Table S2. Fatty acid composition (μg g^−1^) of red king crab eggs in crabs from hard- and soft-bottom habitats in Dalnezelenetskaya Bay, July 2016.
