# Supplementary material for: Fatty Acids in the Eggs of Red King Crabs from the Barents Sea
Source: Animals (Basel). 2024 Jan 22;14(2):348. doi: 10.3390/ani14020348 (PMC10812456; doi:10.3390/ani14020348)
Supplement: Supplementary file 1 [file animals-14-00348-s001.zip › animals-2792080-supplementary.pdf]

Table S1. Fatty acid composition (μg g^{-1}) of red king crab eggs in crabs with different numbers of injured legs (0, 1, and > 1) in Dalnezelenetskaya Bay, July 2016.

Fatty acid	0				1				> 1			
	X	SE	Min	Max	X	SE	Min	Max	X	SE	Min	Max
C6:0	2.1	0.3	1.1	4.2	1.8	0.1	1.5	2.6	2.2	0.3	1.3	3.3
C8:0	3.3	0.2	1.9	5.3	3.6	0.3	2.6	5.6	4.6	0.2	4.0	5.1
C9:0	3.2	0.2	1.7	5.6	3.4	0.5	1.9	6.7	4.7	0.4	2.9	5.8
C10:0	6.8	0.3	4.5	8.9	7.7	0.7	5.4	11.0	8.2	0.7	6.1	10.5
C11:0	4.2	0.4	1.5	9.1	3.0	0.3	1.8	4.5	4.8	1.1	1.6	8.0
C12:0	143.6	7.8	83.6	234.3	159.0	9.0	101.8	207.4	132.4	9.6	101.7	164.0
C13:0	20.5	1.1	9.3	31.3	23.7	0.8	19.9	26.4	17.5	1.6	13.2	22.7
C14:0	903	45	480	1335	998	34	818	1112	770	76	534	1007
C15:0	453.9	20.7	278.0	686.9	534.2	13.5	466.3	601.8	449.5	20.2	391.3	521.3
C16:0	7601	302	5453	10787	8783	490	7194	12450	8006	356	6961	9595
C17:0	337.1	13.7	242.4	478.0	402.1	11.1	360.1	459.8	357.7	8.4	333.3	388.1
C18:0	2451	104	1572	3216	2686	138	2106	3468	2365	86	2110	2668
C20:0	139.1	9.5	51.0	198.9	138.8	7.7	99.4	166.7	102.5	8.8	87.2	136.0
C21:0	33.7	4.9	7.1	73.0	40.6	8.6	10.7	74.8	26.0	9.3	10.7	70.7
C22:0	28.8	3.0	5.8	59.5	29.8	4.3	15.0	53.8	24.2	4.1	12.0	36.7
C23:0	109.3	19.2	42.6	447.5	124.9	15.9	81.7	251.7	114.3	11.9	72.8	157.0
C24:0	255.8	33.8	91.1	696.6	349.5	48.9	145.2	625.8	227.5	42.3	116.0	380.7
C14:1t	15.2	0.0	15.2	15.2	–	–	–	–	4.1	0.0	4.1	4.1
C14:1C	12.0	1.2	5.1	28.0	11.0	1.0	3.2	13.9	9.8	1.7	6.0	17.0
C15:1	5.6	1.3	0.8	13.5	2.6	1.4	0.9	5.4	3.9	0.8	3.1	4.7
C16:1t	92.2	4.4	63.3	138.5	106.1	3.5	78.1	115.5	97.3	16.5	21.7	132.6
C16:1C	3595	181	2113	5350	3996	253	2844	5142	3559	234	2973	4391
C17:1	6.8	0.7	3.2	12.3	7.4	1.3	2.9	14.6	4.3	0.4	3.1	4.8
C18:1n9t	274.4	10.5	190.5	356.4	316.8	21.2	205.5	412.6	251.9	23.5	138.1	296.0
C18:1n9C	6543	309	4266	9599	7598	538	5506	11360	7068	482	6017	9155
C20:1	1084	65	562	1897	1220	83	890	1816	1033	115	767	1392
C22:1	119.6	9.9	59.1	204.7	105.4	5.1	86.8	144.3	92.6	5.3	74.8	108.3
C24:1	49.6	3.7	24.9	87.0	51.4	3.4	36.2	63.8	44.9	7.2	25.8	74.0
C18:2n6t	115.2	27.6	9.9	411.7	161.3	42.1	31.2	423.5	209.1	67.4	44.1	365.8
C18:2n6C	629	36	336	995	708	30	603	917	580	49	467	785
C18:3n3	338.8	26.3	152.2	604.6	371.3	38.1	231.5	586.9	286.1	38.6	215.1	465.5
C18:3n6	188.8	14.1	98.3	340.8	230.2	23.1	134.1	332.0	161.8	21.1	86.2	244.0
C20:2	625	31	364	913	656	28	516	814	521	31	423	650
C20:3n6	106.6	8.4	56.2	215.5	104.6	6.4	69.7	128.8	89.7	3.3	82.7	100.5
C20:4n6	2626	165	1183	4251	2985	111	2523	3594	2285	231	1672	3032
C22:2	6.0	0.9	1.6	15.1	6.4	1.0	2.8	13.2	9.7	3.1	0.9	22.6
C20:5n3	13299	692	7530	18244	14561	823	11354	18394	11934	897	10126	15234
C22:6n3	7557	426	5007	12040	9241	486	7107	12037	8498	784	7049	12065
C20:3n3	189.5	17.7	60.3	404.2	187.4	10.2	146.7	242.8	129.0	14.6	97.7	195.3
C22:4n6	196.6	13.5	136.1	415.0	208.3	15.0	135.5	285.8	201.5	19.8	146.9	291.8
C22:3n3	5.2	1.1	1.0	15.4	7.0	1.7	0.9	15.7	3.6	1.9	1.0	10.9
C22:5n6	128.3	6.3	83.0	185.3	166.0	9.2	136.8	215.0	135.6	10.3	101.5	168.7
C22:5n3	804	33	566	1237	882	61	646	1216	924	99	568	1162
∑SFA	12639	518	8369	17368	14290	662	11860	18916	12617	501	11296	14614
∑MUFA	11774	550	7304	17069	13412	810	9799	18425	12162	751	10296	15404
∑PUFA	26815	1288	15831	37113	30475	1365	25094	37468	25929	1671	22647	33235
Total	51085	2325	31504	70464	58177	2738	46754	74809	50712	2786	44723	63253

Note. X – mean, SE – standard error, Min – minimum, Max – maximum, SFA – saturated fatty acids, MUFA – monounsaturated fatty acids, PUFA – polyunsaturated fatty acids.

Table S2. Fatty acid composition (μg g^{-1}) of red king crab eggs in crabs from hard and soft-bottom habitats in Dalnezelenetskaya Bay, July 2016.

Fatty acid	Hard bottom				Soft bottom			
	X	SE	Min	Max	X	SE	Min	Max
C6:0	2.0	0.2	1.1	4.2	2.1	0.4	1.4	3.8
C8:0	3.7	0.2	1.9	5.6	3.1	0.3	2.1	4.5
C9:0	3.4	0.2	1.7	6.7	3.9	0.5	1.7	5.8
C10:0	7.4	0.4	4.5	11.0	6.7	0.4	4.8	7.7
C11:0	4.0	0.3	1.5	9.1	4.0	0.7	1.8	6.8
C12:0	149.5	6.6	83.6	234.3	133.0	5.9	109.6	160.6
C13:0	21.4	0.9	9.3	31.3	18.8	1.4	13.2	22.6
C14:0	911.1	38.1	480.2	1334.7	893.3	50.2	676.7	1023.6
C15:0	485.2	16.9	278.0	686.9	437.8	15.1	391.3	529.5
C16:0	8070	283	5453	12450	7683	359	6835	10055
C17:0	364.0	11.7	242.4	478.0	336.1	8.6	313.3	392.5
C18:0	2506	89	1572	3468	2481	101	2215	3069
C20:0	129.8	7.1	51.0	198.9	144.9	13.0	87.2	176.2
C21:0	35.7	4.3	7.1	74.8	29.2	9.4	10.7	73.9
C22:0	26.9	2.4	5.8	59.5	33.7	4.1	18.2	44.5
C23:0	114.5	14.3	42.6	447.5	113.7	18.3	53.1	189.5
C24:0	296.7	29.6	91.1	696.6	203.6	32.5	116.0	343.4
C14:1t	9.6	5.5	4.1	15.2	–	–	–	–
C14:1C	11.6	0.9	3.2	28.0	10.6	1.2	6.0	15.1
C15:1	5.2	1.2	0.8	13.5	3.4	0.1	3.1	3.6
C16:1t	97.6	4.8	21.7	138.5	94.5	2.9	81.5	104.3
C16:1C	3721	155	2113	5350	3611	226	2973	4997
C17:1	6.3	0.6	2.9	14.6	8.1	1.4	4.7	12.3
C18:1n9t	278.2	11.6	138.1	412.6	296.7	13.5	269.9	360.2
C18:1n9C	6989	297	4266	11360	6638	386	6017	9313
C20:1	1133	57	562	1897	1036	68	784	1284
C22:1	106.0	6.0	59.1	200.3	131.1	16.5	77.1	204.7
C24:1	49.6	3.1	24.9	87.0	48.4	3.4	33.8	57.6
C18:2n6t	134.0	22.3	12.7	411.7	165.9	63.9	9.9	423.5
C18:2n6C	661.6	28.7	336.3	995.3	572.4	32.3	467.4	720.8
C18:3n3	352.2	23.4	152.2	604.6	291.3	20.5	215.1	352.5
C18:3n6	196.7	14.0	86.2	340.8	191.7	9.2	169.1	227.5
C20:2	607.6	24.9	363.5	913.0	649.8	36.4	513.0	813.7
C20:3n6	101.7	6.3	56.2	215.5	109.1	7.0	82.7	132.6
C20:4n6	2687.5	127.6	1183.1	4250.5	2596.0	223.7	1671.6	3348.8
C22:2	7.2	0.9	0.9	22.6	5.0	1.5	1.6	13.2
C20:5n3	13433	591	7530	18394	13366	719	10566	16898
C22:6n3	8419	366	5007	12065	7245	570	5715	10710
C20:3n3	179.7	13.1	60.3	404.2	177.2	21.2	97.7	239.1
C22:4n6	202.8	10.8	135.5	415.0	192.6	15.2	155.4	285.8
C22:3n3	6.2	1.0	0.9	15.7	3.2	1.5	1.0	12.9
C22:5n6	141.8	6.5	83.0	215.0	131.9	6.1	117.5	168.3
C22:5n3	825	32	566	1237	916	75	696	1192
∑SFA	13131	449	8369	18916	12903	534	11719	15986
∑MUFA	12392	491	7304	18425	11874	657	10296	16218
∑PUFA	27950	1081	15831	37468	26609	1338	22647	34989
Total	53473	1977	31504	74809	51014	2471	44723	67193

Note. X – mean, SE – standard error, Min – minimum, Max – maximum, SFA – saturated fatty acids, MUFA – monounsaturated fatty acids, PUFA – polyunsaturated fatty acids.

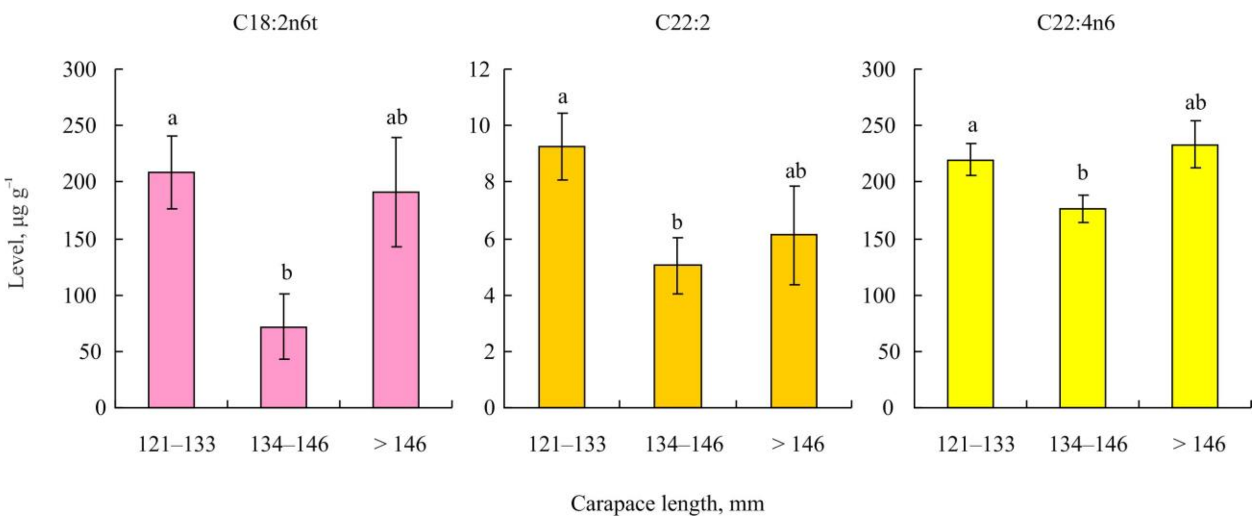

Figure S1. Significant variation in the content of fatty acids in the eggs of female red king crabs of different sizes from the Dalnezelenetskaya Bay, July 2016. Vertical bars show standard errors. Different letters show significant differences between groups.

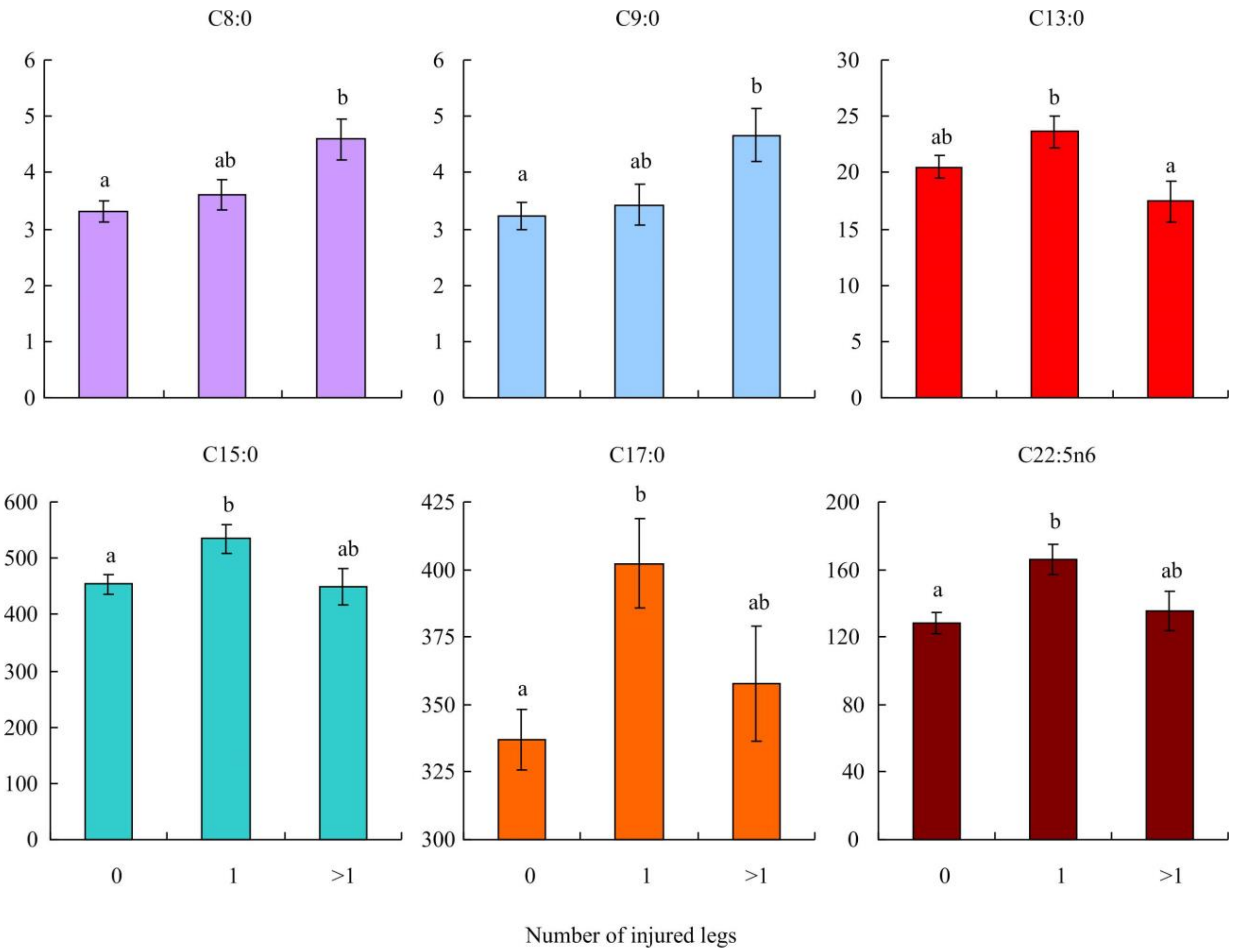

Figure S2. Significant variations in fatty acid content in eggs of female red king crabs with different numbers of injured legs in Dalnezelenetskaya Bay, July 2016. Vertical bars show standard errors. Different letters show significant differences between groups.
